# Autoantibody Epitope Spreading in the Pre-Clinical Phase Predicts Progression to Rheumatoid Arthritis

**DOI:** 10.1371/journal.pone.0035296

**Published:** 2012-05-25

**Authors:** Jeremy Sokolove, Reuven Bromberg, Kevin D. Deane, Lauren J. Lahey, Lezlie A. Derber, Piyanka E. Chandra, Jess D. Edison, William R. Gilliland, Robert J. Tibshirani, Jill M. Norris, V. Michael Holers, William H. Robinson

**Affiliations:** 1 Division of Rheumatology, Department of Medicine, Stanford University School of Medicine, Stanford, California, United States of America; 2 Department of Statistics, Stanford University School of Medicine, Stanford, California, United States of America; 3 Department of Health Research and Policy, Stanford University School of Medicine, Stanford, California, United States of America; 4 VA Palo Alto Health Care System, Palo Alto, California, United States of America; 5 Division of Rheumatology, Department of Medicine, University of Colorado School of Medicine, Anschutz Medical Campus, Aurora, Colorado, United States of America; 6 Walter Reed Army Medical Center, Washington, D.C., United States of America; 7 Department of Epidemiology, Colorado School of Public Health, University of Colorado, Aurora, Colorado, United States of America; University of California San Francisco, United States of America

## Abstract

Rheumatoid arthritis (RA) is a prototypical autoimmune arthritis affecting nearly 1% of the world population and is a significant cause of worldwide disability. Though prior studies have demonstrated the appearance of RA-related autoantibodies years before the onset of clinical RA, the pattern of immunologic events preceding the development of RA remains unclear. To characterize the evolution of the autoantibody response in the preclinical phase of RA, we used a novel multiplex autoantigen array to evaluate development of the anti-citrullinated protein antibodies (ACPA) and to determine if epitope spread correlates with rise in serum cytokines and imminent onset of clinical RA. To do so, we utilized a cohort of 81 patients with clinical RA for whom stored serum was available from 1–12 years prior to disease onset. We evaluated the accumulation of ACPA subtypes over time and correlated this accumulation with elevations in serum cytokines. We then used logistic regression to identify a profile of biomarkers which predicts the imminent onset of clinical RA (defined as within 2 years of testing). We observed a time-dependent expansion of ACPA specificity with the number of ACPA subtypes. At the earliest timepoints, we found autoantibodies targeting several innate immune ligands including citrullinated histones, fibrinogen, and biglycan, thus providing insights into the earliest autoantigen targets and potential mechanisms underlying the onset and development of autoimmunity in RA. Additionally, expansion of the ACPA response strongly predicted elevations in many inflammatory cytokines including TNF-α, IL-6, IL-12p70, and IFN-γ. Thus, we observe that the preclinical phase of RA is characterized by an accumulation of multiple autoantibody specificities reflecting the process of epitope spread. Epitope expansion is closely correlated with the appearance of preclinical inflammation, and we identify a biomarker profile including autoantibodies and cytokines which predicts the imminent onset of clinical arthritis.

## Introduction

Rheumatoid arthritis (RA) is the most common inflammatory arthritis worldwide affecting 0.5–1% of the population. Though RA can present at any age, disease onset typically occurs in the third to eighth decades of life and can cause significant disability, often within the first 1–2 years of clinical disease onset [1]. In most cases, the diagnosis of RA is made clinically and is often delayed by an initial period of non-specific symptoms. It is now generally accepted that there is a brief window of opportunity for early aggressive management of RA and that delay results in increased joint damage and disability [2], in most cases, the diagnosis may be delayed by an initial period of non-specific symptoms. Nearly 70% of cases of established RA are characterized by the presence of autoantibodies, either rheumatoid factor (RF) or antibodies directed against citrullinated proteins (ACPA), of which antibodies to cyclic citrullinated peptides (anti-CCP) are the most specific clinical test currently available [3]–[5]. These antibodies and inflammatory cytokines [6]–[8] are present years prior to the onset of symptoms in RA, suggesting that the autoimmune processes leading to arthritis are present long before overt disease manifestations.

Although the presence of RF and anti-CCP antibodies can aid in making a diagnosis of RA, the sensitivity and specificity of these tests are limited, especially in the early or preclinical period [9]. Use of these markers to predict the time of future onset of clinically-apparent RA is limited by the large time interval during which anti-CCP antibodies and/or RF may be positive prior to the development of clinical RA. The ability to define where in the pre-clinical timecourse an individual patient lies could facilitate not only early identification, but even pre-clinical treatment, in an effort to prevent RA associated morbidity and/or achieve disease prevention. Additionally, the ability to identify and observe the break of immunologic tolerance at the earliest stages of disease could provide significant insights into the pathogenesis of RA and could be used to guide initiation of disease modifying or even tolerizing therapy [10].

In this study, we utilize preclinical RA serum samples obtained from a cohort of military patients who ultimately progressed to clinical RA to characterize autoantibody reactivity and cytokine levels during the pre-clinical period. We demonstrate epitope spread of autoantibody responses and a crescendo of cytokine elevations precede the development of clinical RA. Finally, we identify a panel of autoantibody and cytokine markers that predict the imminent development of clinically active RA.

## Results

### Description of patients and sample characteristics

Baseline demographics are presented in Table 1 and discussed in the methods. The population studied was representative of the military population from which it was drawn with a higher representation of males (60% males) and a slightly younger mean age of clinical onset (age 39). However, no differences were found between genders in outcomes in several stratified anlyses (data not shown). Of 81 subjects included in the analysis, 27 had no autoantibodies detected in their earliest serum specimen and of these all demonstrated at least one autoantibody at a future preclinical RA timepoint. 55 patients were anti-CCP2+ at any timepoint, of which 26 were anti-CCP2+ at outset and 29 converted from anti-CCP2− to anti-CCP2+ during the period of study observation.

**Table 1:** Subject demographics.

	Cases	Controls
**Number**	81	81
**Mean Age at diagnosis**	39.0	39.1
**Years prior to diagnosis of first sample, Mean (STD)**	6.4 (3.7)	6.4 (3.7)
**Male**	49 (60%)	49 (60%)
**Race**	White: 57 (70%)	White: 57 (70%)
	Black: 20 (25%)	Black: 20 (25%)
	Other: 4 (5%)	Other: 4 (5%)
**Samples Per Person, Mean (STD)**	3.5 (1.2)	3.5 (1.2)
**Year range (relative to RA diagnosis)**	−13.6 to 11.7	−13.6 to 11.6
**Anti-CCP2 Ever Positive (at sample time)**	55 (68%)	0 (0%)
**Anti-CCP2 at time of serum sample**	130 (46%)	0 (0%)
**Erosions**	41 (51%) (7 unknown)	

### Accumulation of autoantibodies precedes that of cytokines and chemokines

Figure S1 provides a heatmap presenting the overall results of all markers analyzed, with specimens binned by 2 year intervals preceding RA diagnosis. Two year intervals were chosen to provide as many “windows” as possible into the pre-clinical phase while still allowing a robust number of subjects at each “bin” and to avoid measuring the same subjects in the same “bin” more than once.

Though some specimens are highly reactive more than 8 years prior to the diagnosis of RA, there is a clear accumulation of autoantibody reactivity over time as individuals approach the development of clinical RA. A similar trend is noted for accumulation of cytokines over the pre-clinical period with temporal lag in the development of cytokine elevations relative to elevations in autoantibodies.

### Anti-CCP2 antibody titer is correlated with increasing number of ACPAs and followed by a rise in serum cytokines

**Figure 1:**
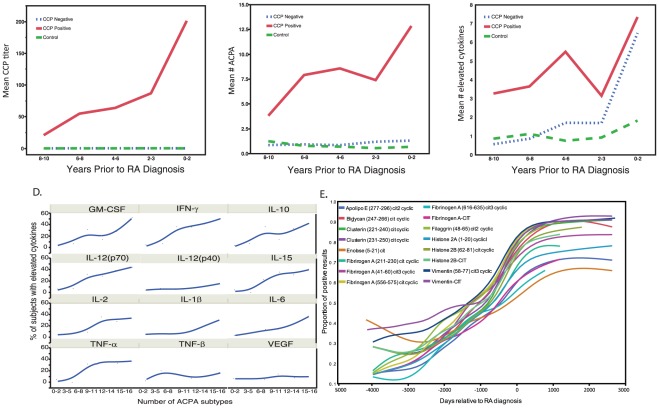
The number of elevated autoantibodies and cytokines increase as individuals in the preclinical period approach the clinical diagnosis of RA. A–C, Mean titers of CCP2 (A), mean total number of ACPAs (B), and mean total number of cytokines (C) were evaluated at each time preclinical timepoint demonstrating a parallel rise in number of ACPA epitopes with rise in anti-CCP2 titer. D, The percent of subjects with elevated levels of each cytokine was evaluated in relation to number of ACPA subtypes present (representative examples of 48 measured cytokines) E, The proportion of subjects positive for each ACPA subtype was evaluated over the preclinical period. The X axis represents days relative to the diagnosis of RA. The Y axis represents the proportion of pre-clinical RA patients with positive value for each marker relative to total number of specimens available for analysis at that timepoint. A–E, Anti-CCP2 antibody titers were measured by CCP2 ELISA (A), ACPA subtypes were measured using a custom multiplex autoantigen bead array, serum cytokine concentrations were measured using commercial bead based multiplex cytokine kits.

The ACPA immune response targets a broad range of citrullinated antigens [11], [12], however, the exact identity of the CCP2 peptide epitopes are currently proprietary and thus unknown. However, the anti-CCP2 test has been demonstrated to captures several overlapping reactivity of ACPA targets [13]. We demonstrate a parallel rise in the number of ACPA subtypes with increasing titer of anti-CCP2 reactivity (Figure 1A and B) suggesting that the ACPA specificities identified likely represent the antibodies bound by CCP2, which represents an artificial mimic of the true citrullinated antigens in RA. Further, our results suggest that the rise in anti-CCP2 titer at least partially represents progressive epitope spread of the autoreactive B cell response targeting citrullinated antigens during the preclinical period. Notably, the rise in both anti-CCP2 antibody titer and number of ACPA subtypes is followed by a rise in serum cytokines (Figure 1C). A very similar rise in hsCRP was noted over the preclinical period as demonstrated in Figure S4A.

### Patterns of autoantibody accumulation

To further evaluate the pattern of autoantibody accumulation over time, we calculated the proportion of subjects positive for each biomarker at each timepoint and generated Kaplan Meier survival curves to compare rates of accumulation (Figure 1E). Though each to a different extent, we observed a gradual increase in rates of positivity for each ACPA approaching clinical diagnosis of RA. Additionally, there were notable differences in the proportion of subjects targeting each antigen as well as the profile of antigens targeted by each individual. Citrullinated histone 2B, citrullinated vimentin, and peptides derived from citrullinated enolase, and fibrinogen represented the most prominent early targets in the pre-clinical period of RA with positive titers in over 25% of subject at 10 year prior to clinical RA onset (Figure 1E). Similar to the results demonstrated in Figure 1B and C, the rise in individual antibodies was followed by the appearance of multiple serum cytokines, again, at a time more proximate to disease onset (Figure S3).

### ACPA epitope spread is associated with the rise in serum cytokines

To assess for associations between the accumulation of autoantibodies and the presence of inflammatory cytokines, we evaluated the correlation between the number of ACPA subtypes with the levels of serum cytokines. Cytokines prominently elevated in association with increased epitope spread included many of those classically implicated in RA pathogenesis [14] including TNF-α, IL-6, IL-12p70, IFN-γ, IL-2, and IL-15 (Figure 1D). Notably, this pattern extends to only a limited number of cytokines suggesting specificity of the cytokine rise associated with expansion of the APCA repertoire. Similar to RA-associated cytokines, level of hsCRP was found to be associated with increasing number of ACPA subtypes.

### ACPA specificities preceding and associated with the development of anti-CCP2 reactivity

**Figure 2:**
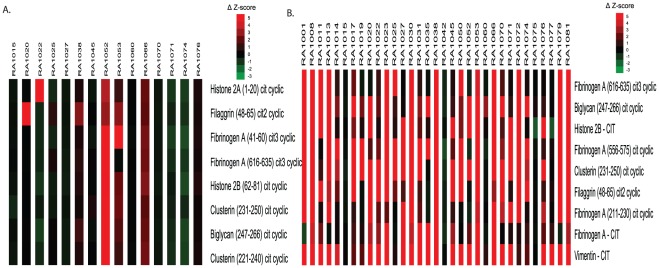
Accumulation of ACPA fine specificity before and concurrent with anti-CCP2 antibody seroconversion. A, Paired SAM analysis was performed on serum specimens from pre-clinical RA patients from whom at least 2 specimens were available prior to anti-CCP2 antibody seroconversion. B, Paired SAM analysis was performed on serum specimens from pre-clinical RA patients for whom a serum specimen was available both prior to and after anti-CCP2 antibody seroconversion. The heatmap represents absolute change in Z-score* from the first to the second timepoint. *Z-score represents the number of standard deviations above or the below the mean level observed in control subjects for each cytokine or autoantibody thus increase in Z-score represents increased change from normal population.

To assess for the development of anti-citrulline reactivity prior to the onset of anti-CCP2 antibody seroconversion, we performed paired SAM analysis of samples derived from individuals who ultimately became anti-CCP2+ but were anti-CCP2− at two timepoints (at least 9 months apart) prior to anti-CCP2 seroconversion. We found evidence of accumulating autoantibody specificities targeting citrullinated histones, fibrinogen, biglycan, and clusterin in subsets of patients preceding the onset of CCP2 reactivity (Figure 2A).

To identify markers associated with the transition from anti-CCP2− to anti-CCP2+, we performed paired analysis to identify differences in autoantibody profiles immediately prior to, and the first timepoint after, anti-CCP2 seroconversion. Our results demonstrate epitope spread of B cell responses against multiple citrullinated antigens in a similar time period to that of anti-CCP2 seroconversion (Figure 2B).

### Multiplex profiles predict imminent development of RA

**Figure 3:**
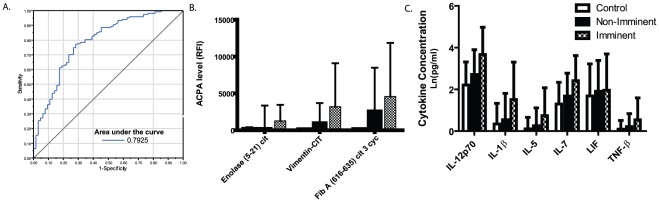
Prediction of imminent RA using multiplex biomarkers. Multiple logistic regression was performed to identify markers from the reduced set of 21 antibodies and 38 cytokines which could classify pre-clinical RA subjects as being within 2 years of the onset of clinical RA. 5-fold cross validation was performed and common markers selected for final validation. A, Demonstrated is a receiver operating characteristic (ROC) curve using the panel of markers listed in table 3. B–C, Mean and standard deviation of values for each individual autoantibody (B) or cytokine (C) contributing to prediction of imminent onset RA as measured among controls, those RA patients at a timepoint greater than 2 years prior to RA onset, or those within 2 years of clinical RA onset.

The ability to predict when an individual will develop clinical RA could facilitate early intervention, and perhaps even prevention, of RA. Using our panel of biomarkers, we applied multiple logistic regression analysis to preclinical-RA patients to determine when a subject/sample was within two years of clinical RA (imminent RA) or at a timepoint not yet within two years of clinical RA. Given the prolonged duration of anti-CCP2 antibody positivity (approximately 6 years [5]) in the preclinical phase, this marker offered minimal predictive utility for imminent onset of clinical RA. However a panel of autoantibodies and cytokines derived from our array displayed a moderate sensitivity (58.2%) and but significant specificity (87%) for identifying patients who were within 2 years of clinical RA onset (Table 2 and Figure 3A). This profile includes autoantibodies targeting epitopes derived from citrullinated fibrinogen (Fibrinogen A 616–635 cit 3 cyc) as well as citrullinated enolase (Enolase 5–21 cit) and whole citrullinated vimentin. Cytokines identifies as predictors included IL-12(p70), IL-1β, IL-5, IL-7, LIF, and TNF-β When each biomarker was evaluated individually, wide deviation limited predictive ability (Figure 3B, and figure S2C), however a use of a multiplex profile of biomarkers predicted imminent onset of RA (Table 2 and Figure 3A). Notably, several ACPA, become positive on only 50–70% of cases (for example, enolase 5–21 cit became positive in approximately 60% of cases) thus identifying one cause for imperfect sensitivity while at the same time demonstrating the specificity of the chosen markers.

**Table 2:** Multiplex predictors of imminent RA diagnosis.

	Sensitivity		Specificity		PPV		NPV	
**Imminent RA Biomarker profile**	58.2%	(32/55)	87.0%	(92/106)	70.0%	(32/46)	80%	(92/115)

## Discussion

In this study we use multiplex autoantibody and cytokine analyses of serum samples to evaluate RA-related autoimmunity and inflammation in the preclinical period. We identified the presence of anti-citrulline autoimmunity years before clinical diagnosis, and demonstrate that preclinical epitope spreading of ACPA responses is associated with the emergence of subclinical inflammation and ultimately, the onset of clinical RA. There was a crescendo in the development of elevated levels of serum autoantibodies as well as cytokines as individuals approached the development of clinical RA. These data demonstrate that epitope spreading of anti-citrulline B cell autoimmunity occurs concurrently with increases in anti-CCP2 antibody reactivity, and that accumulation of anti-citrulline reactivities likely represents the preponderance of what is measured clinically as the anti-CCP2 antibody response. We found evidence of autoantibody targeting of several citrullinated proteins including histones, fibrinogen, and biglycan at the earliest measured timepoints and well prior to development of anti-CCP2 positivity. Early targeting of these innate immune ligands provides potential insights into the mechanisms underlying the initiation of autoimmunity in RA (discussed below). Additionally, we demonstrate a successive accumulation of anti-citrulline autoimmunity before, during, and after the appearance of anti-CCP2 antibody reactivity. Finally, we identify a profile of ACPA subtypes in combination serum cytokines which identify pre-disease patients who were within 2 years of clinical RA onset. Though it is not clear that there is a period when RA is uniquely sensitive to therapy, or that individuals can be treated in a manner to regain tolerance to critical self antigens. However, the ability to identify individuals in the earliest phases of disease would provide a potential window of opportunity for studies to investigate very early or even preclinical intervention.

Similar to work in systemic lupus [15], [16], several of the earliest and most prominent autoantigens identified in our study have been demonstrated to possess innate immune stimulatory capacity including fibrinogen [17], biglycan [18], and histones [19]. Work by our group [20] has demonstrated the ability of citrullinated fibrinogen-containing immune complexes to co-stimulate macrophage cytokine production, and others [19], [21], [22] have demonstrated the ability of DNA or RNA-containing immune complexes to stimulate B-cell proliferation and dendritic cell cytokines activation via Toll-like receptors. Thus, the presence of citrullinated antigen-immune complex with the potential co-stimulate B cells and/or macrophage further implies that a break in tolerance to citrulline modified proteins may function in the initiation, propagation, and effecter phases of clinical RA development.

Several previous studies have examined preclinical autoantibodies including anti-CCP antibodies [3]–[5], certain anti-citrullinated protein antibodies [23], [24], and rheumatoid factor [25]–[27] and others have demonstrated a preclinical rise in serum cytokines or other inflammatory markers [7], [8], [28]. Similar to our result, Jorgensen, et al [29], [30] observed that the presence of anti-CCP antibodies and RF preceded the elevation serum cytokines and Nielen, at al. [31] found that the appearance of anti-CCP and RF antibodies shortly preceded a rise in CRP levels. Interestingly, Aho et al [32], were unable to show preclinical elevations of CRP, even in individuals with longstanding elevation of RF, though they did not measure serum cytokines and it is possible that there is a narrower window needed to observe a rise in preclinical CRP [31].

Several groups have determined the sensitivity and specificity of anti-CCP and RF antibodies in preclinical subjects for the ultimate diagnosis of RA [3], [4]. Here we demonstrate the utility of ACPA and cytokine profiling to identify individuals at high risk for imminent progression to RA. Our study demonstrates the use of a panel of ACPA specific for the citrullinated epitopes present on our arrays as well as cytokines, in a multiplex fashion, to improve preclinical diagnostic accuracy and to narrow the temporal period during which an at-risk individual will develop RA. These profiles could be applied to identify individuals in populations at high risk for developing, including first degree relatives [33], [34] and those with early musculoskeletal compaints [35]. The benefit to such knowledge includes the potential to identify individuals with imminent or very early RA and thereby provide the opportunity to intervene at a time either before, or very early in clinical RA development.

Several groups have attempted to predict which individuals will progress from early undifferentiated arthritis (UA) to RA [36]–[38], and found that the presence of RF and anti-CCP antibodies to be highly associated with progression to clinical RA. Similarly, van der Woude and colleagues, using a panel of 5 citrullinated peptides, observed an increase in the number of epitopes recognized over the period preceding RA onset as well as in those progressing from UA to RA [39]. Additionally, a recent interventional study demonstrated that methotrexate therapy during the period of UA significantly increased the proportion of patients entering clinical remission, though this benefit was limited to patients with anti-CCP antibody reactivity [40]. Thus use of a more sensitive APCA profile could allow identification of other who would benefit from such intervention and potentially even earlier in the pre-RA period. Preclinical autoantibody accumulation has similarly been associated with development of systemic lupus [16], multiple sclerosis [41], and type 1 diabetes. Thus, multiplex platforms such as ours may be amenable to translation to other autoimmune conditions and similar techniques to identify multiplex profiles may be utilized for prediction of disease onset or outcome in other disease types.

Autoantibody profiling of sera derived from animal models of RA and multiple sclerosis (MS), in which a single protein is used as the immunogen, have demonstrated a pattern of intra- and inter-molecular epitope spreading leading up to and through clinical disease onset [42], and autoantibody profiles have been used to guide tolerizing therapies in the same models [10], [43]. Thus profiling of autoantibody fine specificity has the potential to be applied to individuals in the earliest phases of clinical arthritis, a time period during which they would potentially respond to specific tolerizing therapies.

Our results support a model in which, in the predisposed host (due to genetics and/or environment), there is an initial break in tolerance to naturally occurring citrullinated antigen(s) such as histones [44] or fibrinogen [45] which is followed by inter- and intra-molecular epitope spreading. Our finding that autoantibody targeting of citrullinated histones, fibrinogen, and biglycan, all molecules that bind innate immune receptors, supports a model in which citrullination of these antigens could co-ligate receptors on B cells to trigger an autoantibody response against these molecules. Given the ability of autoadjuvant-containing immune complexes to stimulate B cells in systemic lupus [19], and the ability of citrullinated fibrinogen immune complexes to co-stimulate macrophage [46], an analogous mechanism could activate B cells specific for citrullinated histones or fibrinogen to produce and thus initiate the process of epitope spreading to a broad range of citrullinated antigens. We hypothesize that at a threshold in the number of ACPA and/or development of a critical specificity profile there is initiation and/or propagation of a subclinical inflammatory response manifest by a rise in serum cytokines. Whether this is of articular origin is uncertain, as the relationships between the likely extra-articular site of initial autoantibody generation in RA to inflammation in the joint is currently unknown. In addition, the increase in inflammation may also reflect alterations in effector functions of ACPA to allow more effective complement/FcR engagement, or IgE-mediated mast cell activation. Ultimately, the autoimmune response reaches an inflammatory threshold characteristic of clinically apparent RA. Of interest, a nadir was noted in ACPA and cytokines approximately 4 and 3 years prior to diagnosis, respectively (Figure 1B,C). It is interesting to hypothesize that this may be related to a regulatory process which attempts to, and in the case of those who ultimately develop RA, fails to prevent the development of clinical autoimmunity. It does not seem related to processing or storage as samples in this preclinical timeframe as these samples were collected and processed over a wide time-frame relative to each other.

Limitations of our study include use of synthetic peptides which were citrullinated at sites of potential modification, only some of which have been confirmed *in vivo*. Similarly, cyclization may provide not only increased signal to noise but also reactivity to epitopes not naturally targeted in RA patients. Additionally, it is possible that, in certain autoantigens, 3-dimensional epitopes are critical to autoantibody recognition, and thus we may miss reactivity against antigens that are represented on the array only by peptides or for which conjugation to beads results in conformational hindrance. Our arrays include antigens implicated in the literature and identified through proteomic screens performed in our laboratory and in the laboratories of our collaborators [47], and thus may omit critical autoantigens or antigenic modifications [48] not previously described. This is especially problematic if we seek to extrapolate our results to seronegative (i.e., true ACPA negative) samples, for which the target antigens have not been identified. Additionally, the measurement of only total IgG ACPA and not IgG isotypes, IgA or IgM may miss signal related to maturation of the adaptive immune response [24] or similarly miss a signal from the recently observed IgE ACPA response [49]. Our study population represents a relatively younger, healthier, population (reflecting a typical military population). Thus, our findings may overestimate specificity for RA in these subjects, especially when compared to an older and the more heavily female population that would be encountered when screening a more general population at risk for RA. We were also limited by the broad variability in the timing of serum sampling within our cohort. Given the lack of consistent sampling intervals, in many cases we were unable to define the exact time of seroconversion for specific epitopes and thus likely overestimate the proximity to disease onset of their appearance. We suspect this spread in sampling, in addition to patient variability, limited our ability to define patterns of autoantibody fine specificity, especially in the earliest phase of autoimmunity when the break in immune tolerance first occurs. Similarly, there is likely wide variety in the timing of presentation with “clinical RA” both due to to patient delay as well as different thresholds to assign diagnosis by physicians. It is also notable that time of diagnosis could be substantially earlier if the new 2010 ACR/EULAR RA criteria [50] were utilized rather than 1987 guidelines [51]. However, it is useful to point out that the median time of onset of symptoms was ∼six months prior to diagnosis, and in no instance where it could be assessed did symptoms precede the presence of autoantibodies [5].

Finally, it is clear that despite attempts to remove redundant biomarkers, there was still significant overlap in markers used for our final analysis. Given the high degree of correlation among ACPA reactivities and between many cytokines, entry of these markers into our stepwise regression analysis could have potentially yielded other closely-related but valid profiles with similar predictive ability. Thus it is difficult to definitively establish that our biomarker panel has unique predictive ability rather than being representative of a critical accumulation of ACPA and inflammatory mediators. In conclusion, we demonstrate that progression from preclinical autoimmunity to clinical RA is associated with (i) progressive epitope spreading of the ACPA response, as evidenced by the targeting of additional citrullinated epitopes (both intramolecular and intermolecular spreading), (ii) inflammation, as evidenced by increases in blood cytokine levels, and (iii) that citrullinated protein epitopes derived from fibrinogen, histones and vimentin are targeted at the earliest observable break in tolerance to citrullinated antigens in at least a subset of individuals with preclinical RA. Together, these results further suggest that the development and epitope spreading of anti-citrullinated protein autoimmunity plays a central role in the initiation of RA-associated autoimmunity as well as progression of individuals from preclinical to clinical RA. Finally, further development and validation of preclinical ACPA and cytokine profiling provides the opportunity to identify and potentially intervene in those individuals at highest risk for imminent development of clinical RA.

## Methods

### Subjects

The Department of Defense Serum Repository (DoDSR) was established in 1989 and has stored serum samples obtained from the United States Armed Forces personnel on enlistment, deployment, and, on average, every other year thereafter. Samples are stored in a central repository at −30 deg C.

For this analysis, the study group included members of the US Military assigned to the North Atlantic Medical Region who were seen at the Walter Reed Army Medical Center (WRAMC) Rheumatology Clinic between 1989–2003 and were diagnosed with RA. Of 156 new onset cases of RA, 83 were found to have prediagnosis serum samples available in the DoDSR, with 66 (80%) having two or more prediagnosis serum samples, and 39 (47%) having four prediagnosis samples. Eighty-three control subjects without RA matched to cases based on age, gender, race, and region of assignment, and time of serum sampling were selected from the DoDSR. Of the 83 cases with pre-diagnosis serum samples, 81 met at least four of seven criteria for RA based on the 1987 American Rheumatism Association's revised criteria and two cases met three of seven criteria and were considered to have RA by board-certified rheumatologists.

Two cases and respective controls were excluded for lack of available sample within the immediate 10 years preceding clinical diagnosis. Five samples (3 cases, 2 controls) were excluded due to fluorescent values of over 800 on reagent blank beads, a control procedure utilized to identify non-specific elevations in fluorescence. Demographic information was obtained by chart review at the WRAMC (Table 1). The mean age at diagnosis was 39 and mean time of the earliest serum sample was 6.4 year before diagnosis, with a mean of 3.5 samples per patient available for analysis. Reflecting a military population, the cohort was 60% male.

Investigators at Stanford University were blinded to group assignments at the time of antibody and cytokine profiling. After the autoantibody and cytokine testing was completed, the coding key was provided to link the serum samples to subject data.

The study protocol was approved by the respective Institutional Review Boards at the Walter Reed Army Medical Center (WRAMC), the University of Colorado, and Stanford University. Due to the retrospective nature of this serum repository cohort, informed consent from subjects was not possible and thus the need for informed consent for the retrospective serum retrieval protocol was waived by the ethics committees at WRAMC and University of Colorado. All investigations conformed to the principles expressed in the 1975 Declaration of Helsinki [52].

### Clinical autoantibody assays

Anti-CCP2 antibodies assays were performed at the University of Colorado School of Medicine in the Rheumatology Clinical Research Laboratory using a CCP2 ELISA kit (Diastat, Axis-Shield Diagnostics.) as previously described [5]. For anti-CCP2 testing, based on the cut-off level established by the manufacturers, a level of >5 units was considered positive.

### Multiplex cytokine assays

We performed multiplex analysis of 48 cytokines and chemokines (listed in Table S2) using the Bio-Plex™ bead array system as recommended by the manufacturer. Using manufacturer provided reagents, we evaluated several commercial reagents to suppress the effect of heterophilic antibodies and, similar to the observation of others [8] and in contrast to our previous observations with other commercial kits [53], found that none consistently suppressed cytokine values in those specimens containing RF relative to those without RF. Data processing was performed using Bio-Plex manager software version 4.4.1 and serum concentrations were interpolated from standard curves for each respective cytokine. All cytokines were evaluated using Cox proportional hazards regression and only cytokines with a significant hazard ratio (P<0.05) between cases and controls were using in the final analyses (Table S1). This protocol and data generated were MAIME compliant and were deposited in the Gene Expression Omnibus Repository (accession number GSE32021; http://www.ncbi.nlm.nih.gov/geo/query/acc.cgi?acc=GSE32021).

### Multiplex autoantibody assays

We developed a novel multiplex platform for analysis of 17 autoantibodies targeting putative RA associated autoantigens (and 3 native protein controls; Table S1) using a custom Bio-Plex™ bead-based autoantibody assays in which antigens are conjugated to spectrally-distinct beads (described above). Briefly, protein antigens were coupled to beads using N-hydroxysuccinimide ester chemistry, and peptide antigens synthesized with C- terminal biotin (by Fmoc chemistry) and coupled to avidin-coated beads. Pooled beads were mixed with serum samples and diluents and incubated at room temperature. After washing, anti-human IgG antibody conjugated to phycoerythrin (PE) was added to the dyed beads and incubated at room temperature. After another wash, the bead mixture was passed through a laser detector (Luminex 200) that identifies beads based on the fluorescence of the dyes. The amount of antibody bound to each bead was determined by the fluorescence of PE.

For all analyses, three internal controls consisting of sera pooled from individuals with low, intermediate, or high autoantibody reactivity were run in parallel to assure reproducibility. . Each bead mixture contained serum verification beads and reagent blank beads (RBB) used to verify the addition of serum and the absence of significant nonspecific binding, respectively. This assay yielded highly reproducible results with 7 fold intra-assay coefficient of variance of 0.9–6.8% and 14 fold inter-assay CV ranging from 5.9–19.5% (over 90% of beads yielding inter-assay variances of <12%; Table S2.) This protocol and data generated were MAIME compliant and were deposited in the Gene Expression Omnibus Repository (accession number GSE32021; http://www.ncbi.nlm.nih.gov/geo/query/acc.cgi?acc=GSE32021).

### Statistical analysis

For descriptive analyses of the biomarker data as continuous variables, raw data was normalized by calculating a Z-score using the formula: ((observed value)-(mean value of control patients))/(standard deviation). Z-scores were used because of the differing magnitudes and variances between levels of individual cytokines and autoantibodies; without standardization the analyses are dominated by numerical differences rather than comparative differences in cytokine or autoantibody level. Normalized values thus represent the number of standard deviation above or the below the mean level seen in control subjects for each cytokine or autoantibody. For predictive modeling, data were cube root transformed and positive markers defined as 3 standard deviations above the mean levels of that marker in all normal control samples.

In some patients, markers calculated to be positive at an early timepoint were subsequently calculated to be negative (this was observed for less than 10% of measurements), however most ultimately reverted back to positive at a future timepoint. For descriptive analyses, if a test was positive once, then reverted to negative without a subsequent positive, the test was counted as negative. Likewise, if a positive test became negative, but was then positive on the subsequent measure, then all interval results were considered positive. For modeling analyses no such correction was applied.

For timepoint comparisons, Z-normalized values were analyzed by SAM (Significance Analysis of Microarrays Version 3.08) [54]. Output was sorted based on false discovery rates (FDRs) in order to identify antigens with the greatest differences in autoantibody reactivity between RA patients at different time points. We used hierarchical clustering software Cluster® 3.0 to arrange the SAM results according to similarities among autoantibody specificities, and results were displayed using Java Treeview® (Version 1.1.3).

### Predictive modeling of imminent onset RA

To define a profile of markers which is associated with imminent onset of RA, logistic regression analysis was performed (JMP, SAS Institute Inc.) between groups defined as (i) imminent RA (within 2 years of RA diagnosis) or (ii) non-imminent RA. Modeling was performed on cube root normalized values of cytokine concentrations or autoantibody florescent intensities dichotomized as positive or negative (as above). Nested models were constructed, 20% of samples were held for the test set, and 5 fold internal cross-validation used for marker selection from the training set. To improve diagnostic homogeneity, our prediction model was limited to RA subjects whom were ultimately anti-CCP2+ at time of diagnosis (n = 55 subjects, 68%; 438 serum samples of which 390 were classified as “non-imminent”, and 48 were classified as “imminent”).

## Supporting Information

Table S1 **List of antigens and cytokines/chemokines analyzed.** Final antigen list was selected as described in the methods section. Cytokines/chemokines were evaluated using a commercial 27-plex and 21-plex cytokine/chemokine kit (Bio-Rad Laboratories). For the all predictive analyses only analytes which significantly different between RA cases and healthy matched controls were used for statistical modeling.
(DOC)

Table S2 **Representative intra-assay and inter-assay coefficients of variance (CV) on the BioPlex antigen array platform.** To validate the reliability of the novel bead-based antigen array 7-fold intra-assay and 14-fold inter-assay CV was calculated.
(DOC)

Figure S1 **Heatmap of autoantibody and cytokine levels during the preclinical period in individuals that developed RA.** Results from preclinical and post-diagnosis serum specimens were binned by timepoints relative to the time of diagnosis of clinical RA, and the matched samples from health controls binned binned in a parallel manner. Results are expressed as *Z-normalized values for each biomarker, and the scalebar represents Z-normalized value. *Z-normalized values represent the number of standard deviations above or the below the mean level observed in control subjects for each cytokine or autoantibody. (Ag = Antigen, Ck = Cytokine)
(EPS)

Figure S2 **Alternative demonstration of values of biomarkers contributing to imminent RA biomarker profile.** Scatter plots demonstrate actual values and range of individual subjects for each biomarker comprising the imminent RA biomarker profile. Imminent onset RA is defined as onset within 2 years of serum sampling.
(EPS)

Figure S3 **The proportion of subjects positive for each cytokine/chemokine evaluated over the preclinical period.** The X axis represents days relative to the diagnosis of RA. The Y axis represents the proportion of pre-clinical RA patients with positive value for each marker relative to total number of specimens available for analysis at that timepoint. Note, cytokines with no observed rise are represented as incomplete lines to allow visualization of rising curves.
(PDF)

Figure S4 **Evaluation of preclinical rise in hsCRP.** A, Mean level of hsCRP evaluated at each preclinical timepoint demonstrates a rise in concentration during the preclinical period (as observed for ACPA and cytokine number as well as anti-CCP2 titer demonstrated in Figures 1 A, B, C). B, Percent of subjects with elevated levels of hsCRP was evaluated in relation to number of ACPA subtypes present.
(EPS)
