# Correction: Autoantibody Epitope Spreading in the Pre-Clinical Phase Predicts Progression to Rheumatoid Arthritis

**DOI:** 10.1371/annotation/2e462817-ab93-4d78-95a4-1d8b9d172971

**Published:** 2012-08-28

**Authors:** Jeremy Sokolove, Reuven Bromberg, Kevin D. Deane, Lauren J. Lahey, Lezlie A. Derber, Piyanka E. Chandra, Jess D. Edison, William R. Gilliland, Robert J. Tibshirani, Jill M. Norris, V. Michael Holers, William H. Robinson

The affiliations for the fourth and sixth authors were incorrect. Lauren J. Lahey and Piyanka E. Chandra are not affiliated with 1 and 2, but 1 and 4.

There was an error in the legend for Figure 3. The correct legend for Figure 3 is: Multiple logistic regression was performed to identify markers from the reduced set of 21 antibodies and 38 cytokines which could classify pre-clinical RA subjects as being within 2 years of the onset of clinical RA. 5-fold cross validation was performed and common markers selected for final validation. A, Demonstrated is a receiver operating characteristic (ROC) curve using the panel of markers listed in figure 3a and b. B-C, Mean and standard deviation of values for each individual autoantibody (B) or cytokine (C) contributing to prediction of imminent onset RA as measured among controls, those RA patients at a timepoint greater than 2 years prior to RA onset, or those within 2 years of clinical RA onset. 

