# Supplementary material for: Autoantibody Epitope Spreading in the Pre-Clinical Phase Predicts Progression to Rheumatoid Arthritis
Source: PLoS One. 2012 May 25;7(5):e35296. doi: 10.1371/journal.pone.0035296 (PMC3360701; doi:10.1371/journal.pone.0035296)
Supplement: Table S1 — List of antigens and cytokines/chemokines analyzed. Final antigen list was selected as described in the methods section. Cytokines/chemokines were evaluated using a commercial 27-plex and 21-plex cytokine/chemokine kit (Bio-Rad Laboratories). For the all predictive analyses only analytes which significantly different between RA cases and healthy matched controls were used for statistical modeling. (DOC) [file pone.0035296.s001.doc]

*Supplemental Table 1. List of antigens and cytokines analyzed*

| **Antigens** | **Cytokines/Chemokines used in model** | **Cytokines/chemokines not used in model*** |
| --- | --- | --- |
| **Peptide antigens:**   |  | |  | | | | --- | --- | --- | --- | --- | | Fibrinogen A (41-60) cit3 cyclic | | | | | | Fibrinogen A (211-230) cit cyclic | | | | | | Fibrinogen A (556-575) cit cyclic | | | | | | Fibrinogen A (616-635) cit3 cyclic | | | | | | Filaggrin (48-65) cit2 cyclic | | | | | | Biglycan (247-266) cit cyclic | | | | | | Clusterin (221-240) cit cyclic | | | | | | Clusterin (231-250) cit cyclic | | | | | | Histone 2A (1-20 cit) cyclic  Histone 2B (62-81) cit cyclic | | | | | | Apolipoprotein E (277-296) cit2 cyclic | | | | | | Enolase (5-21) cit | | | |  | | Clusterin (221-240) cit | | | | | | Vimentin (58-77) cit3 cyclic | | |  | | | **Protein antigens:** | | |  | | | Fibrinogen A-CIT | | |  | | | Histone 2B-CIT | | |  | | | Vimentin-CIT | | |  | | | Fibrinogen A | | | |  | | Histones 2B | | | |  | | Vimentin |  | | |  | | | IL-1β | | --- | | IL-1ra | | IL-2 | | IL-4 | | IL-5 | | IL-6 | | IL-7 | | IL-8 | | IL-9 | | IL-10 | | IL-12(p70) | | IL-13 | | IL-15 | | Eotaxin | | FGF basic | | G-CSF | | GM-CSF | | IFN-γ | | IP-10 | | MCP-1 | | MIP-1α | | MIP-1β | | TNF-α | | VEGF | | IL-1α | | IL-2Ra | | IL-3 | | IL-12 | | IL-16(p40)  LIF | | MCP-3 | | MIF | | MIG | | β-NGF | | SDF-1α | | TNF-β | | IFN-α2 | | IL-18  GRO-α  HGF  M-CSF  SCF  SCGF-β  TRAIL  IL-17  PDGF-BB  RANTES |

* Cytokines/chemokines not used due to lack of significant differences between cases and controls
