# Supplementary material for: Autoantibody Epitope Spreading in the Pre-Clinical Phase Predicts Progression to Rheumatoid Arthritis
Source: PLoS One. 2012 May 25;7(5):e35296. doi: 10.1371/journal.pone.0035296 (PMC3360701; doi:10.1371/journal.pone.0035296)
Supplement: Table S2 — Representative intra-assay and inter-assay coefficients of variance (CV) on the BioPlex antigen array platform. To validate the reliability of the novel bead-based antigen array 7-fold intra-assay and 14-fold inter-assay CV was calculated. (DOC) [file pone.0035296.s002.doc]

Supplemental Table 2. Representative intra-assay and inter-assay coefficients of variance (CV) on the BioPlex antigen array platform.

|  | **Intra-assay CV (n = 7)** | **Inter-assay CV (n=14)** |
| --- | --- | --- |
| Vimentin-CIT | 0.9 – 2.0% | 6.2 – 7.5% |
| Histone 2B-CIT | 1.1 – 3.4% | 5.9 – 11.6% |
| Fibrinogen A-CIT | 1.9 – 2.7% | 10.9 – 16.9% |
| Vimentin (58-77) cit3 | 6 – 6.8% | 15.1 – 19.5% |
| Fibromodulin (295-313) | 3.0 – 3.9% | 6.0 – 10.9% |
