# Supplementary figures and images for: Autoantibody Epitope Spreading in the Pre-Clinical Phase Predicts Progression to Rheumatoid Arthritis
Source: PLoS One. 2012 May 25;7(5):e35296. doi: 10.1371/journal.pone.0035296 (PMC3360701; doi:10.1371/journal.pone.0035296)

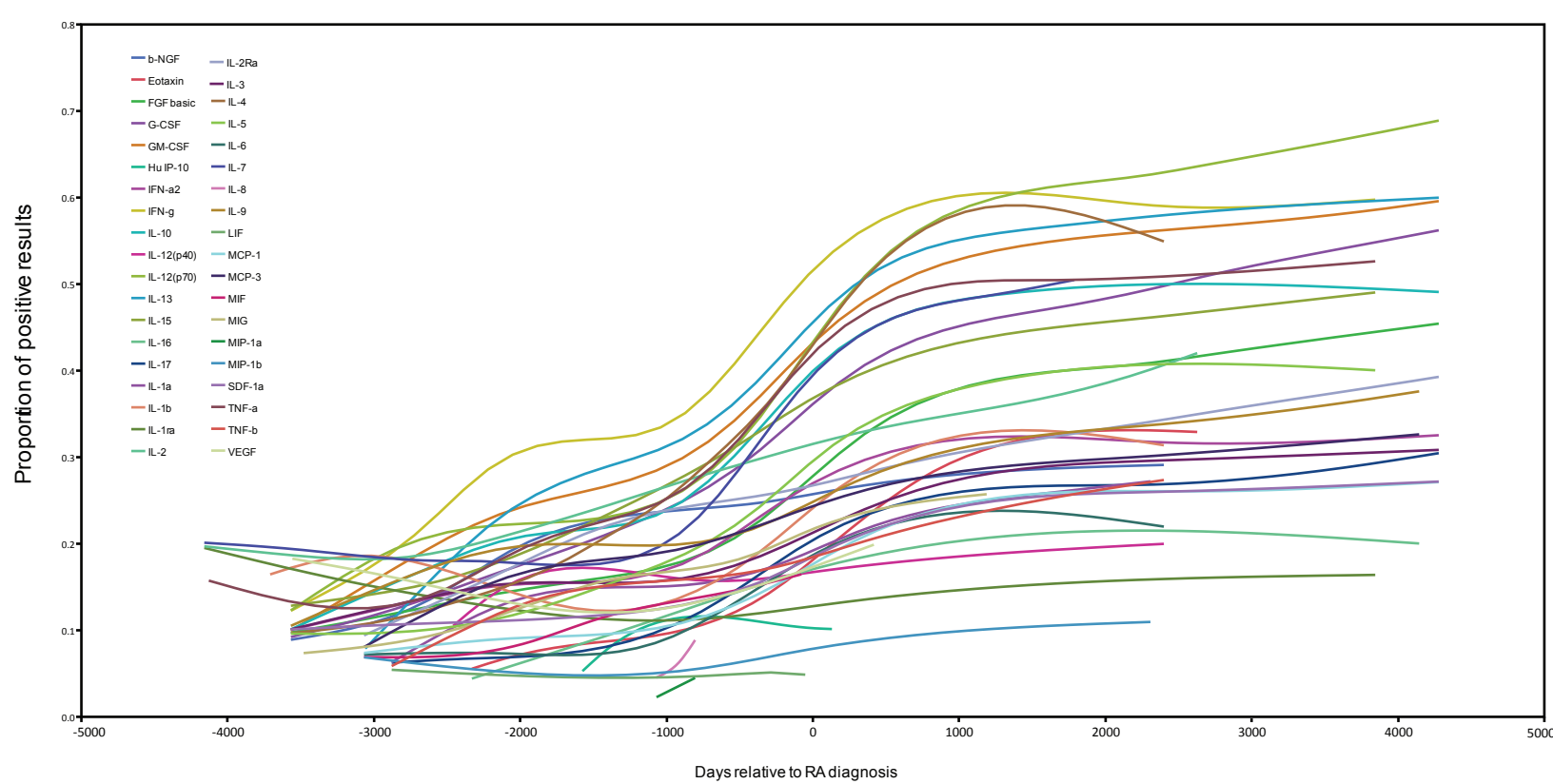

Supplement: Figure S3 — The proportion of subjects positive for each cytokine/chemokine evaluated over the preclinical period. The X axis represents days relative to the diagnosis of RA. The Y axis represents the proportion of pre-clinical RA patients with positive value for each marker relative to total number of specimens available for analysis at that timepoint. Note, cytokines with no observed rise are represented as incomplete lines to allow visualization of rising curves. (PDF) [file pone.0035296.s005.pdf]
